# Why do we need imagination–reality monitoring?

**DOI:** 10.1093/nc/niag048

**Published:** 2026-08-20

**Authors:** Nadine Dijkstra, Stephen M Fleming, Nicholas Shea

**Affiliations:** Department of Imaging Neuroscience, University College London, 12 Queen Square, WC1 3AR London, United Kingdom; Department of Experimental Psychology, University College London, 26 Bedford Way, WC1H 0AP London, United Kingdom; Max Planck UCL Centre for Computational Psychiatry and Ageing Research, 10-12 Russel Square, University College London, WC1B 5EH London, United Kingdom; Institute of Philosophy, School of Advanced Study, Senate House Malet Street, WC1E 7HU, University of London, United Kingdom

**Keywords:** imagery, metacognition, reality monitoring

## Abstract

The ability to imagine scenarios decoupled from the immediate environment supports a wide range of cognitive functions, such as planning, memory, and decision making. However, this increase in cognitive sophistication comes with a potential cost of mistaking simulation for reality. Here, we provide a functional analysis of the imagination–reality problem and argue that the specific way that imagination is used to guide behaviour puts unique constraints on the ability to distinguish it from reality. Our analysis illuminates the space of possible solutions supporting the ability to separate imagination from reality and offers a roadmap for future empirical research.

Imagination allows us to simulate novel scenarios and make inferences about potential futures. For example, you might use your imagination to decide whether the couch that you are planning to buy will fit through your front door (Kind 2017), or whether novel combinations of food, such as tea-jelly, would taste good or bad (Barron *et al*. 2013). In order for these simulations to be useful and guide behaviour appropriately, they need to accurately incorporate perceptual and causal knowledge of the world. In other words, they need to generate reliable and accurate answers to the question ‘what would happen if X?’ (Moulton and Kosslyn 2009).

Maintaining a clear distinction between imagination and perception is essential for adaptive behaviour: if imagining a tiger leads you to believe that a real tiger is standing in front you, you might be frightened, run away and generally act in maladaptive ways. Consequently, systematic confusions between imagination and perception are a debilitating characteristic of various psychiatric and neurological disorders, such as schizophrenia, PTSD, dementia, and certain forms of Parkinson’s disease (Pearson and Westbrook 2017).

Take the example of using your imagination to determine whether a couch fits through your door frame. As a result of a wealth of past experience, the brain contains rich sensorimotor knowledge of how objects move when we act on them. Relying on these inferential connections, we can imagine what the couch would look like under various rotations. Running a simulation in this sensorimotor system supports inferences about the perceptual consequences of moving the couch through the door frame in different possible orientations, i.e. imagining the couch to ‘see’ whether it could fit. However, running these simulations should not make you believe that you have already brought your couch inside.

Similarly, using your imagination to determine the tastiness of tea-jelly requires the information about the flavour of tea and the consistency of jelly to be combined. Crucially, it then depends on using connections from gustatory experience to form evaluative appraisals to infer the value of the new combination, i.e. you imagine tea-jelly in order to ‘taste’ whether you like it. Again, imagining tea-jelly should be distinguished from actually tasting tea-jelly and deciding that you have already had your share of sweets for the day.

In this paper, we argue that the specific way in which imagination is used to guide behaviour creates constraints on how these simulations can be distinguished from perception of external reality.

There are several ways in which the brain could use imagination to guide behaviour. One option is to copy all sensorimotor knowledge into a separate ‘imagination’ system and then perform simulations within this system. This dual system implementation of imagination would put no constraints on distinguishing it from reality, as completely separate systems are used. However, in order to use this imagination system for simulations that lead to correct inferences about the real world, it would have to be a complete duplicate of the perceptual system, including its learnt inferential connections, even though it is solely used to simulate alternative scenarios. These types of dual system architectures are theoretically possible but would be a very inefficient use of neural resources.

A related option would be to temporarily copy only the perceptual representations that are needed for a specific simulation into a separate simulation system, thereby reducing the requirement for a full duplication of perceptual space. Again, because a separate system is used for simulation and perception, there is no danger of confusing imagination and reality. However, a copy of a perceptual representation does not suffice, on its own, to draw inferential consequences. The learnt connections are needed as well. By analogy, I could copy a representation of the game state in a video game by taking a photograph of the video screen. But the photo in my camera is no use for simulating what happens next. The camera does not have access to the underlying structure to run a simulation.

Another option is to reuse existing perceptual systems for imagination. Utilizing the same system ensures that the resulting perceptual predictions are based on the same statistical associations that were learned by perceiving the outside world, and, therefore, have maximum predictive validity. In line with this idea, there is now substantial evidence from human neuroimaging that perceptual brain systems are indeed re-activated during imagery (Kreiman *et al*. 2000; Dijkstra *et al*. 2019; Pearson 2019), further supported by recent single-cell recordings (Wadia *et al*. 2026). However, a same-system solution leads to an imagination–reality problem: how do we know at any given moment whether the system’s activity reflects reality or imagination? Next, we outline possible solutions to this problem and discuss what constraints they put on the types of simulations that can be run.

One possible solution is that imagination only recruits specific subcomponents of the perception system. Imagination–reality monitoring could then be implemented by tracking involvement of the parts of the system not involved in imagination. For example, it has been proposed that imagination often only relies on spatial schemas, or physics engines, without reactivating all relevant sensory details of the imagined scenarios (Bigelow *et al*. 2022; Balaban and Ullman 2025). In other words, we do not need to simulate all sensory details for all tasks. For example, when imagining moving a couch through a door to decide whether it will fit, the colour of the couch is irrelevant. If imagination only ever relied on spatial schemas, the imagination–reality problem could easily be solved by tracking whether fine-grained sensory details are activated or not.

However, simulating sensory detail is important for fine-grained generalization (Lau 2022). For instance, if we experience food poisoning after a particular meal, it may be adaptive to generalize this to other foodstuffs that taste or look similar at a fine-grained perceptual level, rather than to represent only that a certain general category of food is to be avoided. Projecting such memories back into sensory space can enable the efficient generalization of prior experience to experiences that share qualitative similarities (Dunsmoor and Murphy 2015; Fleming and Shea 2024). Furthermore, even when imagining the couch, sensory details like its colour might still be included in the simulation, even if this is not required for the task at hand. In this case, tracking the activation of sensory detail would not distinguish imagination from reality.

Rather than providing a categorical distinction between imagination and reality, sensory richness might allow for a graded reality inference. Even in fine-grained generalizations, some sensory details are often still left out. For example, when imagining tea-jelly to decide whether it will taste good, imagining the plate that holds the tea-jelly, or even the shape of the tea-jelly, is unnecessary. In line with this, imagination is generally described as being less vivid than perception (Koenig-Robert and Pearson 2021) and recent empirical studies have shown that subjective vividness ratings of imagined objects are predictive of the likelihood that participants mistake them for perception (Kwon *et al*. 2022; Dijkstra and Fleming 2023; Dijkstra *et al*. 2025). One important focus for future research is to characterize how sensory richness is used for imagination–reality monitoring and what counts as ‘rich enough’ to cross a reality threshold (Dijkstra and Fleming 2023).

Another possible solution is that imagination is accompanied by an additional signature that is not present in perception, or *vice versa*. One candidate signature is top-down control (Johnson *et al*. 1993; Haggard 2017; Dijkstra *et al*. 2022). This would mean that simulations that are accompanied by strong top-down control can be distinguished from reality by virtue of monitoring this control signal. However, not all sensory activation during imagination is accompanied by top-down control, sometimes images pop into our mind involuntarily (Pearson and Westbrook 2017). Furthermore, top-down control of sensory activation can also be employed during perception in the form of attention. Therefore, similar to sensory richness, top-down control might not categorically separate imagination and reality but might instead provide evidence for graded reality inferences. Therefore, another important focus for future research is to investigate whether and in which circumstances top-down control informs imagination–reality monitoring, and how this interacts with the influence of sensory richness on reality inferences.

Taken together, neither using only a sub-set of the perceptual system nor tracking an imagination-unique signature seems to categorically distinguish all instances of imagination and perception. Therefore, imagination–reality monitoring requires a flexible evaluation process that can adjust to changing task conditions. This process would evaluate and integrate different factors such as sensory richness and controllability and output a reality judgement. It has been suggested that prefrontal circuits specialized in metacognition are recruited to gate perceptual activity into experience by determining whether the activity reflects reality rather than noise (Fleming 2020; Lau 2022). One possibility is that these same circuits are also recruited to determine whether sensory activity reflects reality or imagination (Simons *et al*. 2017; Dijkstra *et al*. 2022; Lau 2022). In line with this idea, recent studies show that neurons in prefrontal cortex show both shared and distinct codes for memory and perception (Tanigawa et al. 2022, Mendoza-Halliday and Martinez-Trujillo 2017). Dedicated research programs are needed to further elucidate the neural mechanisms supporting reality inferences.

To conclude, in order for imagination to adaptively guide behaviour, it needs to access learned sensorimotor information. Accessing this information by reusing the perceptual system is efficient but leads to an imagination–reality source monitoring problem. This problem could be solved by using only part of the perceptual system or by including an additional unique imagination signature, but neither solution is sufficient in all imagination tasks. Future empirical research should investigate how task demands constrain the way that the imagination–reality problem is solved.

## Data Availability

There are no new data associated with this article.
